# Short-Term Re-intervention of Endovascular Abdominal Aortic Aneurysm Repair

**DOI:** 10.7759/cureus.16810

**Published:** 2021-08-01

**Authors:** Hesham Soliman, Mohamed Elkorety, Mohamed Abouelazayem, Girish Girish

**Affiliations:** 1 Department of General Surgery, Kings College NHS Foundation Trust, London, GBR; 2 Department of General Surgery, West Hertfordshire Hospitals NHS Trust, Watford, GBR; 3 Department of General Surgery, St George’s University Hospitals NHS Foundation Trust, London, GBR

**Keywords:** evar, abdominal aortic aneurysm, endovascular, re-intervention, endo vascular intervention

## Abstract

Background and aim

Endovascular aneurysm repair (EVAR) has revolutionized the management of abdominal aortic aneurysm (AAA). The re-intervention rate following EVAR has been a subject of debate in many studies. The study aims to evaluate the short-term outcomes in terms of the early (four-year) re-intervention rate following EVAR at our centre and compare it to the average re-intervention rate of the main studies assessed by the National Institute of Health and Care Excellence (NICE).

Methods

The EVAR procedures performed over two years (2015 and 2016) were retrieved using the operation codes. The clinical portal and PACS systems were used to review the discharge summaries, clinic and multidisciplinary team (MDT) letters, as well as the scans and interventional radiology procedures to assess the patients’ adherence to follow-up and identify any re-intervention procedure done to correct underlying problems related to the EVAR performed. Patients who switched their follow-up to another hospital were contacted and interviewed about any re-intervention undergone.

Results

A total of 108 patients underwent EVAR during the two-year study period. Twenty EVAR-related re-interventions (18.5%) were recorded, irrespective of the cause or the type of intervention. This is slightly higher than the average rate by NICE (16.89%). Type 1 endoleak represented the leading cause for re-intervention (30%). Most of the cases of re-intervention were done endovascularly (60%). Forty-five percent of the patients had a re-intervention during the first year and 35% in the third year.

Conclusion

This study shows that although our re-intervention rate following EVAR was slightly higher than the international average, EVAR is still a safe method for the repair of AAA with relatively low peri-operative morbidity and mortality. However, long-term follow-up of these patients is mandatory as re-interventions are frequently required. Nonetheless, the majority of re-interventions can be done with minimal morbidity to the patient.

## Introduction

The first endovascular repair of infra-renal abdominal aortic aneurysm (AAA), which was reported by Parodi et al. in 1991, revolutionized the management of AAA [1]. The endovascular grafts used nowadays have significantly improved from the first generation ones, and continue to evolve to offer easier delivery and deployment while maintaining better seal fixation over the long term. It is well established that endovascular aneurysm repair (EVAR) offers a much greater early postoperative outcome when compared to the traditional open surgical repair, in the form of decreased intraoperative bleeding, reducing both intensive care unit and overall hospital stay, as well as early postoperative mortality. However, these benefits come at the expense of a much higher incidence of re-intervention rate, hence the need for close and prolonged follow-up [2]. This is certainly more important now, given the latest developments from the National Institute for Health and Care Excellence (NICE).

The study aims to evaluate the short-term outcomes in terms of the early (four-year) re-intervention rate following EVAR at our centre and compare it with the average re-intervention rate of the main studies assessed in the evidence review of AAA: diagnosis and management by NICE guidelines. A secondary aim is to evaluate the different devices used in EVAR regarding the rate and causes of re-intervention. The four-yearly re-intervention rate following EVAR in ACE (Anevrysme de l'aorte abdominale, Chirurgie versus Endoprothese), EVAR-1 and OVER (Open versus endovascular repair) trials is 16.89%; these trials combined a total of 1220 EVAR cases [3].

## Materials and methods

The EVAR procedures performed from 1st of January 2015 till the 31st of December 2016 were retrieved using the operation codes. The clinical portal and PACS systems were used to review the discharge summaries, clinic and multidisciplinary team (MDT) letters, as well as the scans and interventional radiology procedures to assess the patients’ adherence to follow-up and identify any re-intervention procedure done to correct underlying problems related to the EVAR performed. Patients who switched their follow-up to another hospital were contacted and interviewed about any re-intervention undergone. 

## Results

A total of 108 patients underwent EVAR during the two-year study period. The study included 93 males (86.1%) and 15 female (13.9%) patients. The average age of the patients was 75.6 years ranging from 57 to 89 years old. Only two repairs were done urgently for ruptured AAA, while the rest were done as an elective procedure following detailed discussion in the vascular MDT. The patients' co-morbidities are illustrated in Table 1.

**Table 1:** Number and percentages of co-morbidities of the study patients

Condition	Cases	Percentage
Hypertension	71	65.7%
Diabetes	19	17.6%
Chronic kidney disease	23	21.3%
Coronary artery disease	62	57.4%
Chronic obstructive pulmonary disease	34	31.5%
Smoking	63	58.3%
Hyperlipidemia	67	62%
Peripheral arterial disease	42	38.9%

Twenty EVAR-related re-interventions (18.5%) were recorded, irrespective of the cause or the type of intervention. Although 30 patients (27.8%) developed a type of endoleak post-operatively which required close follow-up, only 17 patients (15.7%) had a re-intervention to further investigate or treat this endoleak. Type 1 endoleak represented the main cause for re-intervention (30%), other causes are summarized in Table 2. Most of the cases of re-intervention were done endovascularly (60%) (Table 3).

**Table 2:** Causes of re-intervention and percentages

Cause of intervention	Cases	Percentage
Type I endoleak	6	30%
Type II endoleak	5	25%
Type III endoleak	2	10%
Type V endoleak	2	10%
Migration	2	10%
Acute Lower limb ischemia	3	15%
Total	20	100%

**Table 3:** Types of re-intervention and percentages

Type of intervention	Cases	Percentage
Endovascular	12	60%
Open	6	30%
Laparoscopic	1	5%
Fluoroscopic	1	5%
Total	20	100%

Our experience with the Gore excluder device yielded the best results, as no re-intervention was recorded for the 10 patients who underwent the EVAR using it. Although The Nellix device was one of the devices least used, it showed a high rate of re-intervention at 37.5%, which included postoperative mortality following open repair for Type I endoleak. The re-interventions with the Nellix device were caused by device migration/Type I endoleak, which contributed to half of Type 1 endoleak cases in our study.

The Ovation device was used in just over half of the cases, followed by the Zenith in 18.5%, the re-intervention rates in both are roughly around the percentage of our average re-intervention figure. The Ovation device re-intervention was related in the majority of patients to Type II endoleak; four cases needed either embolization or ligation of the Inferior Mesenteric Artery (IMA) for Type II endoleak. While three cases developed Type I endoleak, which required a cuff/stent, it is worth noting that the aneurysm neck measurements were within the Instructions for Use (IFU). The rest of the cases were divided between revascularization procedures for acute lower limb ischemia and open repair for Type V endoleak. Seventy-five percent of the re-interventions for the Zenith devices were endovascular repair for Type I endoleak which were contributed to short neck (<12 mm), while one case required a revascularization procedure for acute lower limb ischemia owing to extremely tortuous iliac arteries with tight angles. The only re-intervention case related to the Powerlink device recorded was Fluoroscopic guided embolization using Onyx™ for a Type II endoleak. Types and frequency of devices used and re-intervention rate per device are summarized in Table 4. Looking at the timing of the first re-intervention, 45% of the cases had a re-intervention during the first year and 35% in the third year; these are summarized in Table 5.

**Table 4:** Types and frequency of devices used and the related re-intervention incidence Ovation (Endologix LLC, Irvine, CA, USA). Zenith (Cook Medical Inc., Bloomington, IN, USA). Gore Excluder (W. L. Gore & Associates, Inc., Flagstaff, AZ, USA). Nellix (Endologix LLC). Powerlink (Endologix LLC). Endurant II (Medtronic, Minneapolis, MN, USA). AFX (Endologix LLC).

Device	Number and percentages of device use		Number and percentages of re-intervention per device	
	Number	Percentage	Number	Percentage
Ovation	55	50.9%	11	20%
Zenith	20	18.5%	4	20%
Gore Excluder	10	9.3%	0	0%
Nellix	8	7.4%	3	37.5%
Powerlink	8	7.4%	1	12.5%
Endurant II	6	5.6%	1	16.7%
AFX	1	0.9%	0	0%
Total	108	100%	20	100%

**Table 5:** Time of first re-intervention and percentages

Time of 1^st^ re-intervention	Number of cases	percentage
With the same admission	4	20%
Within 3 months after discharge	3	15%
Within 4-12 months after discharge	2	10%
Total first year	9	45%
Second year	0	0%
Third year	7	35%
Fourth year	4	20%

## Discussion

It is evident that we have a slightly higher re-intervention rate than the international average rate, however, most cases were managed with minimally invasive approaches as endovascular or laparoscopic (70%) and less than a third required either open repair or bypass graft (30%). It is noted that eight patients underwent an additional planned procedure at the time of the primary EVAR, either in the form of embolization or a fem-fem crossover bypass. Although it can be a coincidence that only one of these patients underwent a re-intervention (Palmz stent inserted two weeks later for Type I endoleak), it is encouraged to extensively assess the scans prior to the primary EVAR to ascertain the requirement for an additional procedure done at the same setting to decrease the incidence of re-intervention, and ultimately improve the efficiency of utilization of resources. While rupture of sac following EVAR is considered rare, it has been reported, and so patients who develop persistent endoleak need close and continuous surveillance [4].

Nordon et al. stated that the main bulk of re-interventions are done within one month following EVAR. They stressed the importance of supplementary procedures intra-operatively to evaluate the perfectness of the exclusion of the AAA with the graft, and thus, a precautionary strategy will help to cut down the number of re-interventions and the overall need for close follow-up [5]. This is was not the case with our first-month re-intervention rate (20% of our re-interventions were done in the first month), which is likely due to thorough MDT discussion of the cases at our centre prior to surgery and application of additional procedure if indicated. It is suggested to delay performing the completion angiogram during EVAR until after withdrawal of all sheaths and stiff wires, as they have the potential to mask a kink in the graft, which can only become evident after removal of the wires and sheaths and a tortuous iliac artery reverting back to its natural shape. We have routinely applied this strategy to help achieve adequate positioning of the graft.

The re-intervention rate following EVAR has been a subject of debate in many studies [6-8]; Conrad et al. reported their results of secondary intervention following EVAR at 11% in a cohort study that involved 832 patients. It was stated in this study that the diameter of the AAA prior to repair, as well as the embolization of the IMA, were prognostic factors for re-intervention for endoleak [4]. The continuous possibility for re-interventions following EVAR seems to continue throughout the lifetime of the graft, thus requiring a close and standardized surveillance program. This adds to the already high cost of an EVAR, yet it seems that less than 10% of patients benefit from the surveillance program following EVAR [5].

In contrast to most studies that examined the re-intervention rate after EVAR [2,4,6,7], the most common cause was Type II endoleak. In our study, Type I endoleak made up the majority of the cause for re-intervention (30%). The high incidence could be attributed to the anatomy of the neck of these cases, a third of which had a conical neck and another third had an angulated and short neck (12 mm). Of the 108 cases included in our study, 14 patients (39.4%) developed Type II endoleak; however, only five patients required an intervention to treat this, the rest are under surveillance or the endoleak had resolved by itself. Type I endoleak was recorded in eight patients (7.4%), of which six patients had an intervention to address this. One resolved spontaneously of the remaining two patients, and the other was deemed unfit due to poor general condition. This could be due to surgeons pushing the boundaries of IFU for a device leading to more Type 1 endoleaks, which earlier were otherwise either not treated due to fitness issues or underwent more a complex procedure like fenestrated EVAR.

The gold standard management for Type II endoleak remains a subject of debate. It is the most commonly occurring endoleak in 10% to 25% of patients following EVAR [9-11]. On follow-up, they resolve on their own in almost 75% of the cases [9-11]. Because of this, Type II endoleaks are usually observed, and intervention is only required in cases of sac enlargement. A patent IMA is one of the common causes of persistent Type II endoleak [4]. Some centres advocate the embolization of patent IMA using coils prior to EVAR. Although this has been reported to have been performed with minimal adverse effect, including colon vascularity, it in fact did not prove to reduce the incidence of Type II endoleak [12].

On the other hand, more recent studies like that published by Axelrod et al. reported less persistent endoleaks and a greater shrinkage of sac in patients who underwent preoperative embolization of the IMA when compared to those who did not [13]. In our study, only four patients (3.7%) from 108 patients who underwent EVAR required IMA embolization or ligation within four years. It is not in our current practice to routinely embolize or ligate the IMA prior to EVAR. The efficacy and overall safety of EVAR during the early postoperative period has been well documented in many studies [14,15]. A meta-analysis including 21,178 patients showed that the 30-day mortality was significantly inferior in patients undergoing EVAR when compared with those who underwent open AAA repair (P < 0.001) [16]. This has been proven in our study as the 30-day mortality post-EVAR was nill.

The main principle of EVAR is the total exclusion of the aneurysmal sac and depressurization, to eliminate the risk of aneurysm rupture. It is well documented that the rupture rate following EVAR is minimal; Conrad et al. reviewed 4291 patients who underwent EVAR from the EUROSTAR registry and only identified 34 patients (0.8%) who had a rupture following EVAR [4,17]. The main cause of rupture has been thought to be endoleaks [4]; Types I and III were the most recorded Types [17].

The limitations of this study are primarily being a retrospective study, and variations in case selection and device use as well as follow-up plan by individual surgeons involved in the management of patients, though all cases are discussed in a formal MDT before surgery and any re-intervention.

## Conclusions

This study shows that although our re-intervention rate following EVAR was slightly higher than the international average, EVAR is still a safe method for repairing AAA with relatively low peri-operative morbidity and mortality. However, long-term follow-up of these patients is mandatory as re-interventions are frequently required. Nonetheless, the majority of re-interventions can be done with minimal morbidity to the patient.
